# Celluloid

**Published:** 1883-10

**Authors:** 


### CELLULOID.

A French inventor has succeeded in producing remarkable results by means of celluloid printing plates, both from engravings and type. A copy of the engraving or the type is made with a special cement, which sets in about twenty minutes. A sheet of celluloid is employed to obtain a counter-impression from this, by means of a moderate degree of heat, and this is then prepared by the ordinary means for the press. A celluloid plate can be made in an hour, and one has been subjected to twenty-five thousand impressions without losing its sharpness and peculiar clearness of detail.

